# Supplementary material for: Saccharomyces boulardii (CNCM I-745) improves intestinal damage in sepsis by remodeling intestinal flora structure
Source: Front Cell Infect Microbiol. 2025 Jul 31;15:1602792. doi: 10.3389/fcimb.2025.1602792 (PMC12350329; doi:10.3389/fcimb.2025.1602792)
Supplement: Supplementary file 1 [file DataSheet1.zip › summary/summary/7_Differential_analysis/2_LEfSe_result/Group_DW4_vs_CW4_vs_BW4_vs_AW4_0.05_3.0/LEfSe_Group_DW4_vs_CW4_vs_BW4_vs_AW4.barplot.pdf]

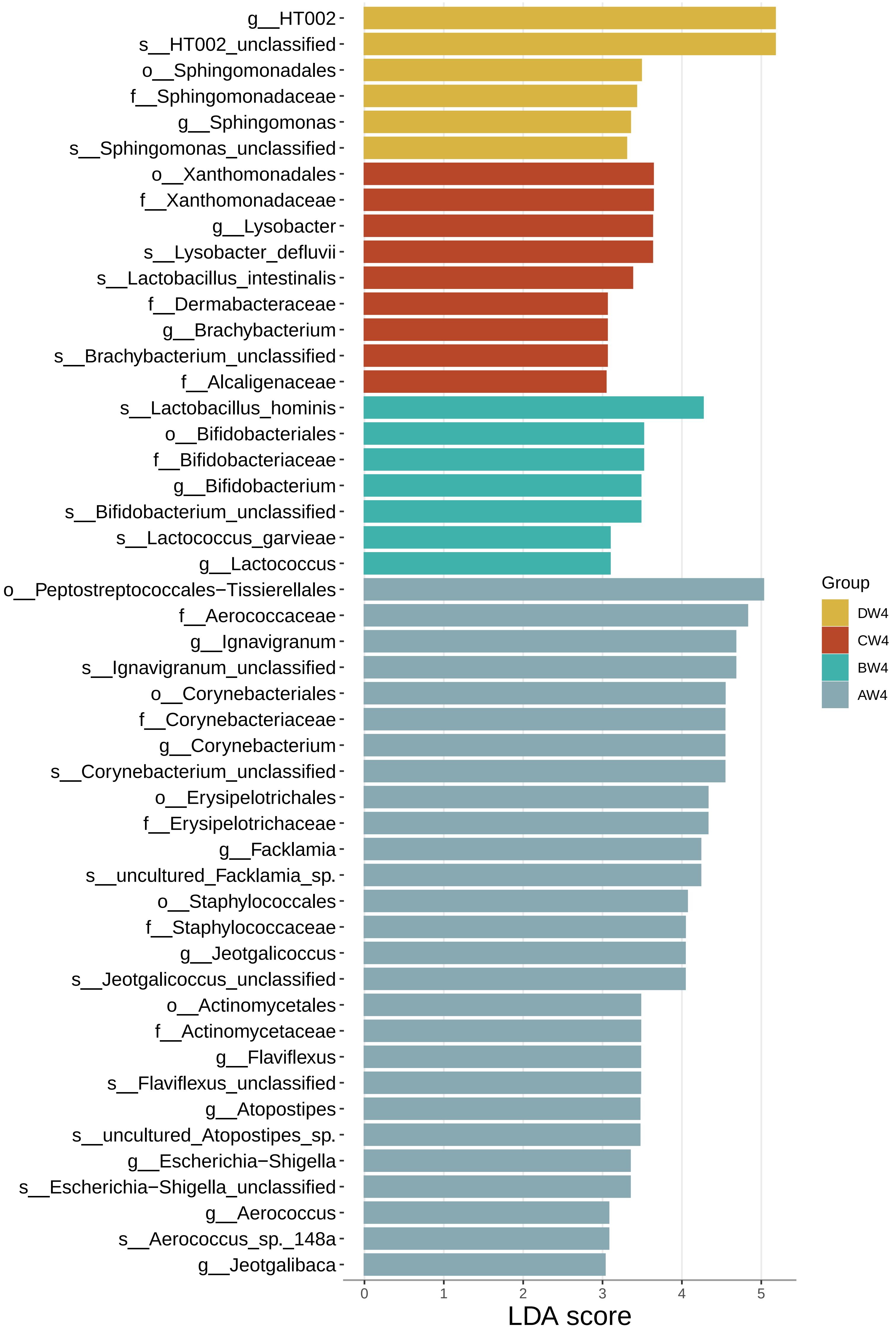
g__HT002
s__HT002_unclassified
o__Sphingomonadales
f__Sphingomonadaceae
g__Sphingomonas
s__Sphingomonas_unclassified
o__Xanthomonadales
f__Xanthomonadaceae
g__Lysobacter
s__Lysobacter_defluvii
s__Lactobacillus_intestinalis
f__Dermabacteraceae
g__Brachybacterium
s__Brachybacterium_unclassified
f__Alcaligenaceae
s__Lactobacillus_hominis
o__Bifidobacteriales
f__Bifidobacteriaceae
g__Bifidobacterium
s__Bifidobacterium_unclassified
s__Lactococcus_garvieae
g__Lactococcus
o__Peptostreptococcales−Tissierellales
f__Aerococcaceae
g__Ignavigranum
s__Ignavigranum_unclassified
o__Corynebacteriales
f__Corynebacteriaceae
g__Corynebacterium
s__Corynebacterium_unclassified
o__Erysipelotrichales
f__Erysipelotrichaceae
g__Facklamia
s__uncultured_Facklamia_sp.
o__Staphylococcales
f__Staphylococcaceae
g__Jeotgalicoccus
s__Jeotgalicoccus_unclassified
o__Actinomycetales
f__Actinomycetaceae
g__Flaviflexus
s__Flaviflexus_unclassified
g__Atopostipes
s__uncultured_Atopostipes_sp.
g__Escherichia−Shigella
s__Escherichia−Shigella_unclassified
g__Aerococcus
s__Aerococcus_sp._148a
g__Jeotgalibaca
0
1
2
3
4
5
LDA score
Group
DW4
CW4
BW4
AW4
